# International women physicians’ perspectives on choosing an academic medicine career

**DOI:** 10.1007/s40037-013-0055-2

**Published:** 2013-04-12

**Authors:** Nicole J. Borges, Amelia C. Grover, Anita M. Navarro, Trisha L. Raque-Bogdan, Caroline Elton

**Affiliations:** 1 Department of Academic Affairs, Wright State University Boonshoft School of Medicine, 290P White Hall, Dayton, OH 45401-0927 USA; 2 Department of Community Health, Wright State University Boonshoft School of Medicine, Dayton, OH USA; 3 Department of Surgery, Virginia Commonwealth University School of Medicine, Richmond, VA USA; 4 Association of American Medical Colleges, Washington, DC USA; 5 College of Education, University of Maryland College Park, College Park, MD USA; 6 London Deanery, London, UK

**Keywords:** Women, Physician, Academic medicine, Career

## Abstract

Concerns about recruiting physicians into academic careers is an international issue. A qualitative study with United States (US) women physicians revealed insights into how, when, and why physicians choose an academic career in medicine. The current study explored international women physicians’ perspectives on their career choice of academic medicine and determined if different themes emerged. We expanded the 2012 study of US women physicians by interviewing women physicians in Canada, Pakistan, Mexico, and Sweden to gain an international perspective on choosing an academic career. Interviews were thematically analyzed against themes identified in the previous study. Based on themes identified in the study of US physicians, qualitative analysis of 7 international women physicians revealed parallel themes for the following areas:

- *Why* academic medicine? *Fit*; *People*; *Aspects of academic health centre environment.*
- *How* the decision to enter academic medicine was made? *Decision*-*making style*; *Emotionality*
- *When* the decision to enter academic medicine was made? *Practising physician; Fellowship; Medical student.*

Work-life balance, choosing academic medicine by default, serendipity, intellectual stimulation, mentors, research and teaching were among the areas specifically highlighted. Conclusion: Parallel themes exist regarding how, why, and when US and international women physicians choose academic medicine as a career path.

## Introduction

Concerns about the difficulty of recruiting high-calibre doctors into academic medicine careers are an international issue. In the United Kingdom (UK), for example, response to this issue led to the formation of a UK-wide national working party chaired by Sir Mark Walport [1]. The resulting report published in 2005 highlighted the significance of three main factors: the lack of a transparent clinical-academic career structure, inflexibility in balancing clinical with academic training, and a shortage of appropriately structured clinical academic posts on completion of training. In the light of the report, the structure of clinical academic training has been revised in the UK, and preliminary evidence suggests that at least in the early years of postgraduate training, the new academic foundation programmes are allowing junior doctors to maintain an interest in pursuing a dual clinical academic career [2].

Within this broader context of problems recruiting a high-quality clinical academic workforce, the specific issue of the under-representation of female clinical academics continues to be of concern. Sandhu et al. [3]. reported that despite the fact that the percentage of female medical students exceeds 60 %, women represented a small proportion of clinical academics, and those women who did follow academic pathways tended to be working at lower grades than men. Reasons identified by these authors included the finding that female academics tended to carry out more teaching than their male counterparts, as well as a lack of opportunity for flexible working patterns within clinical academic careers.

Regarding the literature base related to choice of academic medicine as a career for men and women, two relatively recent reviews of the literature [4, 5] both concluded that a void exists regarding gender as it relates to decisions about choice of academic career in medicine. While the 2006 literature review [4] focused on factors (in general) influencing one’s decision to choose (or not choose) this career path, the more recent review conducted in 2010 [5] specifically explored the question of how, when, and why physicians choose careers in academic medicine [5]. Findings from this literature review [5] promoted in-depth investigation of this issue resulting in a qualitative study with United States (US) women physicians [6]. Results provided insights into how, when, and why women physicians choose an academic career in medicine [6]. Interviews revealed a depth of themes in response to this question. The purpose of the current study was to extend the literature in this area of inquiry by exploring international women physicians’ perspectives on their career choice of academic medicine to determine if different themes emerged across women physicians in different countries.

## Methods

With institutional review board approval, we expanded the January 2012 study of US women physicians [6] by interviewing women physicians in Canada, Pakistan, Mexico, and Sweden to gain an international perspective on choosing an academic career. Purposive sampling was used to identify interviewees. Interviews were 30–45 min in duration and were conducted via the telephone or in person. The interview methods and questions for the current study followed the protocol as outlined in the 2012 study [6, p. 114]. Two authors (NJB and TRB) conducted the interviews, an additional two authors (ACG and AMN) read the transcripts and identified and corroborated themes. Another author (CE) verified the themes. Interviews were thematically analyzed against themes identified in the 2012 study [6].

## Results

The women physicians we interviewed were primarily trained in family or internal medicine (although one specialized in dermatology) with years in academic medicine ranging from 1 to 31 years (average 14). Ethnicity of participants was Pakistani, Caucasian, Latino, and Mexican and participants were equally divided regarding type of medical school attended (public vs private). Based on the themes identified in the study of US physicians [6], qualitative analysis of seven international women physicians revealed parallel themes:

- *Why* academic medicine? *Fit* (i.e., perceive a career path in academic medicine suits them); *People (i.e.,* influence of individuals in decision to pursue academic medicine*)*; *Aspects of academic health centre environment.*
- *How* the decision to enter academic medicine was made? *Decision*-*making styles*; *Emotionality.*
- *When* the decision to enter academic medicine was made? *Practising physician; Fellowship; Medical student*.

In addition to these several parallel themes, the following areas were specifically highlighted by the international sample of interviewees.

*Work-life balance.* The international sample of women physicians in academic medicine often expressed aligning their careers with family considerations. This weighed into choosing the academic medicine path. One interviewee remarked,

I started in academia because I thought it was only at that point that I would not be on night call and would be on some days…wasn’t really much into academia…

*Decisions by default.* Women in the current study expressed choosing academic medicine passively, eliminating other career paths until this one was left or conducting clinical research that brought them to this practice option. This process was not always negative, but incorporated work-life balance considerations and/or other serendipitous events in their paths—as described by one of the women we interviewed,

I got into academic medicine because of circumstances… because of a very hectic clinical routine; I could not carry on my job… I couldn’t do routine… I had to do a job where I could just spend few hours like from 8 to 2 or, 8 to 4.

*Serendipity.* Whether it was encountering a mentor, the way they chose a speciality or some other life event, international women physicians all mentioned some type of chance occurrence that influenced their pathway into academic medicine. In response to the question, when did you decide to pursue a career in academic medicine, one woman physician said,

I don’t think I ever did… It just kind of happened.

*Intellectual stimulation.* This element emerged as the international women physicians expressed enjoying the thinking, writing, and learning that comes with the scholarly activities in academic medicine. One of our respondents remarked,

…my view is… I’m curious, and when there are questions that have never been answered… then I need to answer it.

*Mentors.* This group frequently played a role in the ways the international women physicians configured their careers, and frequently they shared how they had now become mentors. One interviewee said,

My mentor… he basically asked me to come into it [academic medicine]…. Another woman remarked, ‘the school dean… if I have to think about someone, that would be him… because he was there when I finished medical school and he was there also when I entered into residency… so he was the one to whom I talked about the next step.

*Research and teaching.* These areas were most frequently mentioned as the draws to academic medicine and the women in this study expressed not knowing much about academic medicine as students, but learned more through their additional years of training. One of the women physicians we interviewed commented,

It was mainly teaching… I really wanted to be close to the students and the residents… and have the opportunity with them to instill some of the discipline for the higher ethics and medical ethics and moral philosophy… and… about all the things related to medicine…and… also very strong about research… about the conditions… making the diagnosis of the learning environment.’ Whereas another respondent said, ‘…during my internship it became clear that I loved to do academics… mainly teaching with the fellow students and residents and patients… I loved the complexity in the clinical learning environment.

A couple of the women we interviewed were extremely progressive—either doing research or curriculum development in a non-traditional way that involved being on the cutting edge and taking the associated risks because of their passion for their pursuit. This progressiveness may have been necessary to overcome the barriers for women at the time they entered academic medicine as several offered the perspective that there were limited opportunities for women in academic medicine.

Participants also discussed the notion of being perceived as a ‘real’ doctor, that one had to be a practising physician in order to uphold this perception and that academic medicine physicians were not always viewed this way. There seemed to be a dichotomy and they had to reconcile their calling with their interests.

## Conclusion

Despite cultural and training differences, similarities exist regarding how, why and when US and international women physicians chose academic medicine as a career path. The serendipity with which both groups of women physicians ended up in an academic career was readily apparent. Perhaps it is the flexibility in their career path that allows these women physicians to be guided by serendipity. Another recurring theme was the importance of mentorship and the diverse group of role models that helped inspire and guide these women towards academic medicine. Both groups of women physicians were also similar regarding their passion for intellectual stimulation which may serve as a basis for promotion and advancement of women in academic medicine. Gender bias and barriers and the challenge of work life balance was also a consistent theme for both groups of women physicians. Further exploration into the details of these issues would be interesting to investigate, such as how the different systems and structures impact issues such as maternity leave and work hours across countries. An interesting difference between the groups of women physicians was the stronger dichotomy within the international group between clinical work and teaching. In the US there is a growing awareness that an individual’s academic contribution can be more traditional such as research but can also be through clinical service and teaching. Known as the ‘triple threat’ these components comprise the academic system and provide the basis for promotion along faculty rank.

In closing, we would be remiss if we did not acknowledge limitations of the current study. These include language barriers between the interviewees and interviewers and not understanding exactly what the interviewees were expressing at times.
